# Reduction in Acute Ecotoxicity of Paper Mill Effluent by Sequential Application of Xylanase and Laccase

**DOI:** 10.1371/journal.pone.0102581

**Published:** 2014-07-24

**Authors:** Saurabh Sudha Dhiman, Gaurav Garg, Jitender Sharma, Vipin C. Kalia, Yun Chan Kang, Jung-Kul Lee

**Affiliations:** 1 Department of Chemical Engineering, Konkuk University, Gwangjin–Gu, Seoul, Republic of Korea; 2 Institute of SK–KU Biomaterials, Konkuk University, Gwangjin–Gu, Seoul, Republic of Korea; 3 Department of Biotechnology, Kurukshetra University, Kurukshetra, Haryana, India; 4 Department of Biotechnology, Maharishi Markandeshwar University, Mullana-Ambala, Haryana, India; 5 Microbial Biotechnology and Genomics, CSIR-Institute of Genomics and Integrative Biology, Delhi University Campus, Delhi, India; Universidade Nova de Lisboa, Portugal

## Abstract

In order to reduce the ecotoxicity of paper mill, four different enzymatic pretreatment strategies were investigated in comparison to conventional chemical based processes. In strategy I, xylanase-aided pretreatment of pulp was carried out, and in strategy II, xylanase and laccase-mediator systems were used sequentially. Moreover, to compare the efficiency of *Bacillus stearothermophilus* xylanase and *Ceriporiopsis subvermispora* laccase in the reduction of ecotoxicity and pollution, parallel strategies (III and IV) were implemented using commercial enzymes. Conventional C_D_E_OP_D_1_D_2_ (C_D,_ Cl_2_ with ClO_2_; E_OP,_ H_2_O_2_ extraction; D_1_ and D_2,_ ClO_2_) and X/XLC_D_E_OP_D_1_D_2_ (X, xylanase; L, laccase) sequences were employed with non-enzymatic and enzymatic strategies, respectively. Acute toxicity was determined by the extent of inhibition of bioluminescence of *Vibrio fischeri* with different dilutions of the effluent. Two-fold increase was observed in EC_50_ values for strategy I compared to the control process. On the other hand, sequential application of commercial enzymes resulted in higher acute toxicity compared to lab enzymes. In comparison to the control process, strategy II was the most efficient and successfully reduced 60.1 and 25.8% of biological oxygen demand (BOD) and color of effluents, respectively. We report for the first time the comparative analysis of the ecotoxicity of industrial effluents.

## Introduction

In the last two decades, use of enzymes, especially hemicellulases, has revolutionized the pulp and paper industry and provided a glimpse of hope that application of enzymes at various levels can reduce the industrial pollution and effluent’s toxicity. However, the current scenario continues to be challenging because of the high pollution load released by the pulp and paper industries, which are still using chlorine-based bleaching sequences [1]. The conventional bleaching methodology is chemical dependent and energy intensive; therefore, a novel enzymatic treatment with safe level of discharge needs to be developed [2]. Hydrolytic enzymes along with a laccase mediator system (LMS) have been more beneficial in reducing the pollution load of industries compared to other strategies [3]. An N-hydroxy-based synthetic mediator was predominantly used for this purpose [4]. However, toxicity and cost are two of the major hurdles, which hamper the industrial applications of these synthetic mediators. Therefore, the application of natural mediators in LMS is one of the alternatives to overcome these disadvantages, even though their application may cause grafting onto the pulp, an increase in kappa number, and a reduction in brightness of the pulp. Since chemical-intensive conventional strategies and enzymatic processes utilizing synthetic mediators release high levels of toxic compounds into water bodies, the entire processing of the effluents should be characterized to analyze their ecotoxicity and other hazardous properties [5].

It has been observed that pretreatment with xylanase alone cannot reduce the pollution load of pulp and paper industry significantly. Hence, it is believed that a cocktail of two or more enzymes could reduce the release of hazardous materials to safer levels [4]. Although enzymes are effective at the pretreatment level, effluents from the entire process should be analyzed to study the enzymatic after-effects. Few reports deal with the characterization of effluents from enzyme-aided bleaching processes, but without evaluating the interaction of technical parameters at the pretreatment level [2]. Therefore, in the present investigation, the individual and cumulative effects of physical parameters on the efficiency of both xylanase and laccase were optimized and were analyzed using a statistical model.

This is the first ever attempt where ecotoxicity of mixed effluents from the entire bleaching process was characterized using Microtox 81.9% basic toxicity assay method along with the evaluation of the reduction in pollution load in terms of biological oxygen demand (BOD) and color. A schematic study was done with four different strategies by supplementing the conventional bleaching sequence with xylanase from *Bacillus stearothermophilus* SDX and Pulpzyme VLBL (Novozyme, Denmark). In sequential strategies, *Ceriporiopsis subvermispora* laccase and the commercial laccase TM L603P (UK) were used with a natural mediator (syringaldehyde, SA) for the extraction of cellulosic fibers from agro-residual material for paper processing.

## Materials and Methods

### Microbial strains and their maintenance

The bacterial culture for xylanase production was isolated from a compost sample and it was identified by the Institute of Microbial Technology (IMTECH), Chandigarh, India, as *B. stearothermophilus* SDX and strain has been deposited at IMTECH and the accession number is 8508 [6]. A lignin-degrading fungal culture of *C. subvermispora* L-14807 SS-3 was used for laccase production [2].

### Enzyme production, extraction and assay

Xylanase production was studied through two-step statistical (Table S1, S2 in File S1) modeling [7] under solid-state fermentation (SSF) conditions. An Erlenmeyer flask (250 mL) containing 5 g of wheat bran as sole carbon source was moistened with 25 mL of modified Horikoshi medium (pH 8.0) [8]. Sterilized substrate, cooled to room temperature, was inoculated with 15% (v/w) inoculum (18 h culture, ∼3.6×10^6^ cells/mL) and incubated at 37°C in a humidified chamber (relative humidity 60–65%) for 96 h. The flasks were gently tapped intermittently to mix the contents. Colonized solid culture (1 g) was extracted twice with 10 mL of phosphate buffer (10 mM, pH 8.0) through a wet muslin cloth. Xylanase activity was determined through modified Bailey’s method [9] by using 3,5-dinitrosalicylic acid (DNS) reagent [10]. All the parameters related to laccase production were optimized through the conventional “one variable at a time” method under submerged fermentation (SmF) conditions. The crude extract of *C. subvermispora* culture was partially purified using ammonium sulfate fractionation (50–70% cut) and was concentrated with a 30-kDa cutoff membrane filter (Amicon). The observed molecular weight of the laccase was approximately 50 kDa. To obtain the concentrated partially purified enzyme, a membrane with a 30-kDa cutoff was used. The laccase activity was determined according to a previously reported method [11]. Crude extracts of both the strains were assayed for other enzymatic activities viz. cellulase and amylase. Similar to xylanase from *B. stearothermophilus* SDX and laccase from *C. subvermispora*, commercial xylanase and laccase were also characterized.

Estimation of the protein content in all the enzyme samples was determined by Bradford’s method [12]. All the experiments were carried out independently in triplicates and the results are presented as the mean of the three values.

### Screening of natural mediators

Different phenolic compounds such as acetosyringone (AS), coniferyl alcohol (CA), *p*-coumaric acid (PCA), 2,6-dimethoxyphenol (DMP), 2,6-dimethylphenol (DP), guaiacol, 4-hydroxybenzoic acid (HBA), 4-hydroxybenzaldehyde (4-HA), and syringaldehyde (SA) were screened to determine their potential as natural mediators that facilitate decolorization. The potential of natural phenols (50 µM each) was determined using the decolorization assay of 25 µM Azure-B [13]. The catalytic efficiency of natural phenols was compared with that of the synthetic mediator 1-hydroxybenzatriazole (HBT) as a control.

### Enzyme, chemicals and software

Commercial xylanase (Pulpzyme VLBL) and laccase (TM L603P) were kindly provided by Novozyme (Denmark) and Treforest Industrial Estate (Pontypridd, CF375 UD, Wales, United Kingdom), respectively. Agro-residual pulp composed of (w/w) wheat straw (*Triticum aestivum*) (78.8%), sarkanda (*Saccharum spontaneum*) (10.6%), and candy (*Eragrostis* sp.) (10.6%) cooked at 165–175°C for 30 min at a pressure of 7.0–7.5 kg m^–3^, was obtained from an agro-based industry (Trident Paper, Barnala, Punjab) in India. The permanganate number, brightness (%ISO), yellowness, and viscosity (poise) of the unbleached pulp were 7.11±0.96, 42.5±5.6, 15.8±2.6, and 5.18±0.96, respectively. Experimental design was generated using the statistical software “Design-Expert 6.0” developed by Stat-Ease Inc. (Minneapolis, MN, USA) to study the individual and cumulative effect of independent variables on enzyme production and biobleaching.

### Parametric optimization for pretreatment of pulp

Pretreatment of pulp with xylanase and laccase was carried out separately. Different levels of all the independent variables designed through central composite design of response surface methodology (CCD-RSM) are given in Table S3 in File S1. For xylanase-aided pretreatment, 10% (w/v) pulp consistence was used and statistical modeling was applied to four independent variables viz. pH, temperature (T_X_), enzyme dose (ED_X_), and retention time (RT_X,_ Table 1).

**Table 1:** Experimental design (coded variables) and results of CCD-RSM for xylanase-aided pretreatment of pulp.

pH	T_X_^a^	ED_X_^b^	RT_X_^c^	PN_X_^d^		B_X_^e^		Y_X_^f^	
				Actual	Predicted	Actual	Predicted	Actual	Predicted
0	0	0	–α	6.92±1.72	6.82±1.13	43.1±5.7	41.5±4.2	14.2±3.1	14.3±1.5
+1	+1	–1	+1	7.12±1.44	7.31±1.32	48.5±5.7	44.7±4.5	14.8±2.4	15.0±1.6
–α	0	0	0	6.74±1.31	6.85±1.15	44.7±6.4	42.8±4.3	14.2±2.1	14.1±1.5
+1	+1	+1	–1	7.21±1.32	7.09±1.24	39.5±4.5	40.5±4.2	14.5±2.9	14.6±1.5
0	0	0	0	6.58±1.35	6.50±0.92	45.8±2.9	45.8±4.1	13.5±1.2	13.5±1.3
–1	+1	+1	–1	6.74±1.23	6.69±0.82	42.1±5.4	42.7±4.3	13.5±1.9	14.1±1.4
0	0	0	0	6.58±1.24	6.50±0.91	45.8±5.7	45.8±4.5	13.5±3.1	13.5±1.3
0	+α	0	0	6.94±1.22	6.85±0.82	43.4±5.6	43.9±4.1	14.1±2.5	14.4±1.4
0	0	+α	0	6.66±1.35	6.87±0.92	44.5±1.5	41.4±4.4	13.8±3.4	14.3±1.5
0	0	–α	0	7.78±1.33	7.33±1.34	37.2±5.4	39.4±3.1	15.4±1.9	14.7±.1.4
–1	+1	–1	–1	6.73±1.24	6.78±1.25	42.1±6.4	41.0±4.2	13.5±2.3	14.0±1.4
+α	0	0	0	7.62±1.42	7.35±1.24	38.5±9.4	39.5±3.3	16.9±3.1	15.7±1.6
–1	–1	+1	–1	6.97±1.85	6.74±0.92	42.2±4.6	45.5±4.4	14.2±2.4	14.4±1.5
+1	–1	–1	+1	7.24±1.23	7.26±1.12	39.6±2.9	40.3±3.1	14.9±1.2	14.8±1.5
+1	+1	+1	+1	6.98±1.34	6.93±1.11	42.5±4.2	43.3±4.2	14.1±2.6	14.4±1.6
0	0	0	0	6.53±1.22	6.50±0.91	45.8±6.8	45.8±4.3	13.5±2.6	13.9±1.3
–1	–1	+1	+1	7.05±1.25	6.88±0.82	42.2±2.5	43.0±4.4	14.0±3.2	13.9±1.4
0	0	0	0	6.53±1.34	6.50±0.81	45.8±4.1	45.8±4.1	13.5±2.1	13.9±1.3
+1	+1	–1	–1	7.12±1.22	7.28±1.12	39.5±6.8	40.1±3.2	14.3±3.2	14.7±1.5
–1	–1	–1	+1	7.04±1.24	7.16±1.12	42.8±5.7	41.4±4.3	14.1±2.5	14.2±1.5
+1	–1	+1	+1	6.96±1.15	6.88±0.93	39.9±6.8	40.6±4.5	14.6±3.1	14.4±1.5
0	0	0	0	6.58±1.42	6.50±0.81	45.8±5.8	45.8±4.1	13.5±2.5	13.9±1.3
0	–α	0	0	6.99±1.34	6.85±0.81	43.7±5.4	42.3±4.4	14.2±1.3	14.1±1.4
–1	–1	–1	–1	6.82±1.32	6.83±0.82	41.5±4.6	42.1±4.2	14.7±2.5	14.1±1.4
+1	–1	–1	–1	7.01±1.55	7.18±1.22	38.7±3.4	38.1±3.1	14.6±2.2	14.7±1.5
–1	+1	+1	+1	6.94±1.51	6.78±0.91	42.5±4.6	42.7±4.5	14.1±2.3	13.8±1.4
0	0	0	0	6.54±1.63	6.50±0.82	45.8±4.5	45.8±4.1	13.5±2.1	13.9±1.4
–1	+1	–1	+1	7.16±1.24	7.06±1.12	41.2±5.8	42.8±4.2	14.9±3.2	14.3±1.5
+1	–1	+1	–1	6.93±1.62	6.99±1.02	40.5±4.6	40.3±4.2	14.2±3.2	14.8±1.5
0	0	0	+α	7.02±1.14	6.98±0.91	42.8±5.3	43.5±4.2	14.1±2.4	13.9±1.4

a – Temperature (°C); b – Enzyme Dose (U/mg); c – Retention time (min); d – Permanganate Number; e – Brightness (%ISO) of pulp after pretreatment; f – Yellowness (b*) of pulp after pretreatment.

After extensive washing with distilled water, xylanase-treated pulp was subjected to LMS. A broad range of reaction parameters (viz. enzyme dose [ED_L_], mediator concentrations [MC_L_], and retention times [RT_L_]) were optimized through CCD-RSM for the LMS (Table 2). The pulp was again washed thoroughly with distilled water before determining its strength. Similar to xylanase pretreatment, regression equation and responses were determined for the pretreatment of agro-residual pulp with the LMS.

**Table 2:** Experimental design (coded variables) and results of CCD-RSM for laccase-aided pretreatment of pulp.

Run	RT_L_^a^	ED_L_^b^	MC^c^	PN_L_^d^		B_L_^e^		Y_L_^f^	
				Actual	Predicted	Actual	Predicted	Actual	Predicted
1	0	0	0	5.69±0.82	5.23±0.53	47.6±4.1	47.8±4.5	12.8±1.2	12.7±1.1
2	+1	–1	–1	5.68±0.82	5.43±0.52	47.5±4.2	49.2±4.5	11.9±1.4	10.6±1.0
3	–1	+1	+1	5.41±0.61	4.76±0.42	48.2±4.2	47.0±4.5	11.5±2.1	11.5±1.3
4	0	–α	0	5.85±0.73	5.23±0.51	46.8±4.2	48.4±5.4	13.2±1.6	12.9±1.4
5	0	0	0	5.69±0.81	6.10±0.42	47.6±4.4	46.0±4.5	12.8±1.3	12.5±1.2
6	–1	+1	–1	5.89±0.72	5.46±0.51	47.6±4.1	46.7±4.5	13.2±1.1	12.3±1.2
7	–1	–1	–1	5.92±0.74	4.88±0.43	48.1±3.8	47.2±4.5	13.8±1.4	11.4±1.0
8	0	+α	0	5.48±0.72	5.46±0.43	48.6±3.9	46.4±4.6	11.3±1.3	11.7±1.7
9	+1	+1	–1	5.41±0.81	5.95±0.44	48.2±3.6	46.6±5.4	11.6±1.2	12.9±1.2
10	+1	–1	+1	6.52±0.83	4.96±0.61	47.6±4.1	47.4±5.5	11.6±1.4	11.5±1.2
11	+1	+1	+1	5.23±0.83	5.66±0.61	48.9±3.6	46.4±4.5	10.9±1.4	12.3±1.3
12	0	0	0	5.69±0.72	5.23±0.52	47.6±3.9	48.4±4.5	12.8±1.5	12.9±1.8
13	+α	0	0	5.36±0.72	5.22±0.52	48.5±4.1	47.0±4.5	11.5±1.4	11.5±1.3
14	0	0	0	5.67±0.72	6.02±0.52	47.6±3.6	47.2±4.5	12.8±1.5	12.6±1.5
15	–1	–1	+1	6.03±0.71	5.23±0.51	46.7±4.1	47.2±4.5	12.9±1.1	12.2±1.6
16	0	0	0	5.69±0.72	5.23±0.33	47.6±3.6	48.4±4.5	12.8±1.2	12.9±1.2
17	0	0	0	5.69±0.71	5.23±0.41	47.6±4.1	48.4±4.5	12.8±1.3	12.9±1.2
18	–α	0	0	6.24±0.83	5.23±0.52	45.9±3.7	47.1±4.4	13.7±1.2	12.1±1.1
19	0	0	+α	5.37±0.71	4.78±0.43	48.9±3.4	48.9±5.4	10.9±1.1	11.2±1.3
20	0	0	–α	5.15±0.72	5.29±0.62	45.4±3.2	47.6±5.4	13.3±1.2	13.0±1.3

a – Retention Time (min); b – Enzyme Dose (U/mg); c – Mediator Concentration (%); d – Permanganate Number; e – Brightness (%ISO) of pulp after *C. subvermispora* laccase mediated pretreatment; f – Yellowness (b*) of pulp after *C. subvermispora* laccase mediated pretreatment.

### Residual laccase activity

Laccase activity was determined under controlled conditions using 50 g oven-dried (OD) pulp and SA as mediator. Residual laccase activity was determined in the presence and absence of pulp and mediators and was expressed as percentage of initial activity, which was measured at the beginning (zero time) of the incubation time. Experiments were performed using 20 U of laccase and 1.5 mM of mediator.

### Scheme (X/XLC_D_E_OP_D_1_D_2_) for biobleaching

The most extensively used bleaching sequence C_D_E_OP_D_1_D_2_ (C_D,_ chlorine and chlorine dioxide; E_OP,_ extraction with oxygen and hydrogen peroxide; D_1_ and D_2,_ chlorine dioxide) was used as the control. In enzyme-aided strategies, only xylanase-assisted stage (X) and pretreatment with both xylanase and laccase along with natural mediator step (XL) were included prior to the conventional sequence.

Dry plastic bags filled with 50 g of extensively washed OD pulp were subjected to different combinations of reaction parameters as designed using CCD-RSM. During chlorination stage, the consistency of the pulp was maintained at 3% for effective penetration of the fiber by the reaction liquor. Experiments were designed with different doses of Cl_2_ at the C_D_ stage, and a kappa factor of 0.25 was used for calculating the Cl_2_ dose. The E_OP_ stage was carried out in a stainless steel vessel at 70±1°C after adding the required amounts of sodium hydroxide (25 kg/T) and hydrogen peroxide (H_2_O_2_; 5 kg/T). The oxygen pressure was maintained at 5.0 and 2.5 kg/cm^2^ for the initial 30 min and final 90 min, respectively. Final pH in the reaction vessel was maintained by adding dilute H_2_SO_4_ before the addition of ClO_2_.

### FTIR and XRD analysis

Fourier Transformed Infrared Spectroscopy (FTIR) spectra of pulp samples were recorded with a resolution of 4 cm^–1^ over the waves ranging from 4,000 to 400 cm^–1^, using 32 scans per sample. FTIR spectrum was analyzed using the FTIR database.

Crystallinity of cellulose was determined for both untreated and enzymatically treated samples by X-ray diffraction (XRD). After freeze-drying, the samples were analyzed using X-ray diffractometer with an X-ray generator (from 2 to 50 of 2*θ* scattering angle). The crystalline index of cellulose (*Xc*) was calculated from the X-ray diffraction patterns using the following equation:
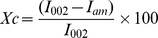
Where I_002_ is the peak intensity from the (0 0 2) lattice plane (2*θ* = 22.3°) and I_am_ is the peak intensity of amorphous phase (2*θ* = 15.7°).

### Analytical studies

Compositional analysis of the agro-residual pulp after pretreatment with different enzyme samples was carried out according to the standard National Renewable Energy Laboratory (NREL) assay procedures [14]. The permanganate number and viscosity were determined according to Technical Association of Pulp and Paper (TAPPI) standards [15]. The brightness, whiteness, optical and color dimensions of hand sheets were also measured (L & W-070; Sweden). The strength of hand sheets was tested according to TAPPI test methods.

Mixed effluents from the different biobleaching strategies have been characterized by analyzing BOD and color following the American Society for Testing and Materials (ASTM) standards, ASTM D-1252-00, and ASTM-1209-00, respectively.

### Toxicity analysis of mixed effluents

Mixed effluents (C_D_, E_OP_, D_1_, and D_2_ in the ratio of 3.25∶1∶1∶1) from all the strategies were assessed using the 81.9% toxicity test protocol with a Microtox Model 500 Analyzer (Modern Water, USA). Different dilutions of each sample were prepared, and the osmolarity of each sample was adjusted with the osmolarity adjusting solution provided by the manufacturer. The endpoint measured by the Microtox assay detects the decrease in the intensity of light emitted by the luminescent marine bacterium *Vibrio fischeri* after 5 and 15 min of exposure. Hence, the EC_50–*5min*_ and EC_50-*15min*_ average values denote the effective concentration (EC) of effluent that caused a 50% reduction in the luminescence of the bacteria [16], [17]. These EC values were calculated using the standard 81.9% toxicity test for each collected effluent sub-sample to determine the acute toxicity of each collected sub-sample. Test results were entered into an Excel worksheet and corrected to normalize the color differences in different treated samples. The unit of acute toxicity (TOU) after 15-min incubation was also calculated for each sample to determine the EC of effluent required to kill 50% of the model organism. The sensitivity of each freeze-dried bacterial sample was periodically checked in our laboratory using zinc sulfate (ZnSO_4_·7H_2_O) as a reference substance.

## Results

### Optimization of enzyme production

All the nutritional and physical parameters affecting the xylanase production were screened through Plackett-Burman (PB) designing (Table S1 in File S1). Based on the regression analysis data of PB design, four significant factors [peptone (*X*_1_), KNO3 (*X*_2_), temperature (*X*_3_) and incubation time (*X*_4_)] were selected and studied at five different levels (Table S2 in File S1) to analyze their individual and cumulative effect on xylanase production through CCD-RSM (Table S3 in File S1). An enhanced (4,220 U/g-dry cell weight) xylanase yield with a specific activity indicative of 597 U/mg-protein after 4 days incubation at 37°C under SSF conditions was achieved for *B. stearothermophilus* SDX by using two-step statistical modeling. The crude extract was free from cellulase and laccase activities and showed very low (12 U/mg-protein) amylase activity.

**Figure 1:**
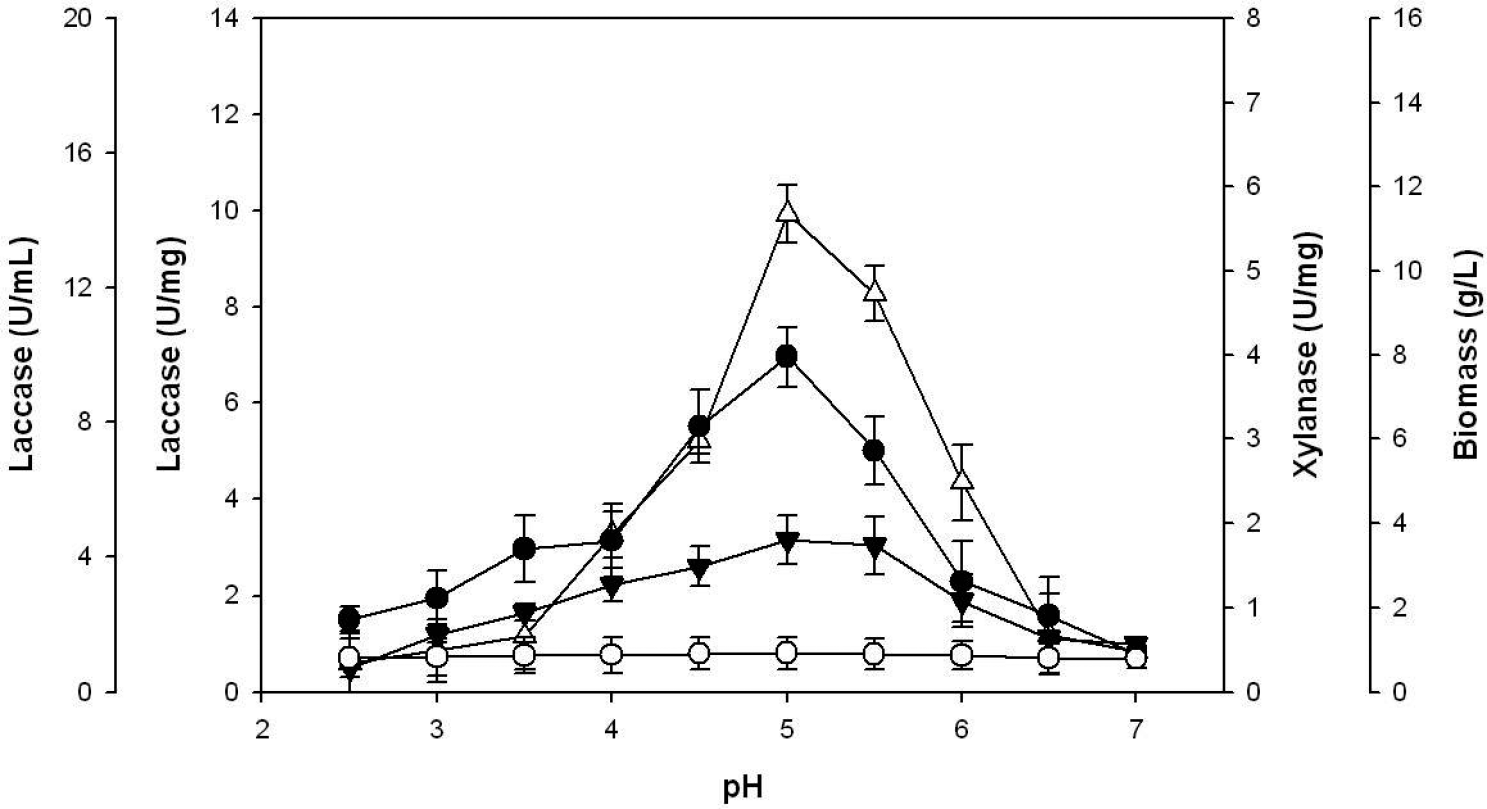
Effect of pH on laccase and xylanase production and growth of fungal mycelium of *C. subvermispora*. *Experiments were carried out at 35°C for 7 days using 2.5% (w/v) of wheat bran in a 250-mL Erlenmeyer flask containing 50 mL of production media. Laccase activity (•); specific activity (▵); xylanase activity (▾); fungal biomass (○).

During laccase production under SmF by *C. subvermispora*, all the parameters were optimized using the “one variable at a time” approach. The highest specific activity of laccase in the crude extract was indicative of 10 U/mg-protein after 7 days incubation at 35°C and pH 5.0 under shaking conditions (50±5 rotations per minute) using 2.5% (w/v) wheat bran (Fig. 1). Crude extract of *C. subvermispora* was free from any cellulase and amylase activity, and contained very less (1.8 U/mg-protein) xylanase activity (Fig. 1). The commercial samples of xylanase and laccase showed specific activities indicative of 498 and 7 U/mg-protein, respectively.

### Selection of natural mediator

*C. subvermispora* and commercial laccase were analyzed with Azure-B dye to verify the mediator capability of all the phenolic compounds. Decolorization of Azure-B was used to verify the potential of natural phenols to behave like mediators in lignin degradation [18]. Among all the tested natural mediators (AS, CA, PCA, DMP, DP, HBA, 4-HA, and SA), only SA showed a significant potential relative to other mediators (Fig. S1 in File S1). In comparison with HBT, AS showed a weak potential, which was reflected by only 5% decolorization (Fig. S1 in File S1).

### Parametric optimization of enzymatic pretreatment

The operational parameters for xylanase-aided pretreatment were optimized through CCD-RSM. A second-order polynomial equation was used to analyze the individual and cumulative effects of four independent variables on the pulp properties (Table 1). Five different levels of each independent variable (Table S4 in File S1) were used to analyze the regression of dependent variables in order to obtain the corresponding responses *Y_1_*, *Y_2_*, and *Y_3_* respectively and facilitate their statistical analysis (Equation S1 in File S1). On the basis of the coefficient of determination (*R^2^ = *0.9029) and an adequate precision (6.446) for the model, 7.5 U of *B. stearothermophilus* xylanase at 60°C and pH 9.0 for 150 min of retention time (i.e., when all the independent variables were at their central point “0”) was found to be the most effective for the pretreatment (Table 1). At optimal conditions, the viscosity of the xylanase produced in the lab and commercial xylanase pretreated pulps were 4.46±0.98 and 4.24±0.84 poise, respectively.

A similar strategy was applied to study the individual and cumulative effects of the laccase dose (ED_L_), natural mediator concentration (MC_L_), and retention time (RT_L_) in the response of the dependent variables of the agro-residual pulp pretreatment (Table 2). Responses of the dependent variables were studied using the second-order polynomial equations (Equation S2 in File S1). The value of *R^2^* indicates that a variation of only 5.41% is possible in the values of the dependent variables. From the statistical modeling it was observed that 20 U (coded value “0”) of *C. subvermispora* laccase with 1.5% of SA concentration (coded value “0”) was the most effective for an incubation period of 225 min (coded value “+α”, Table 2). Other physical parameters (viz. temperature and pH) were optimized by using the “one variable at a time” approach and it was observed that the most suitable conditions for the LMS corresponded to 50°C (Table S5 in File S1) and pH 5.0 (Table S6 in File S1). After sequential pretreatment of the pulp, the viscosity of the enzyme produced in the lab and commercial enzyme pretreated pulps were 4.17±0.76 and 3.89±0.45 poise, respectively. Enzyme supplementation significantly reduced the viscosity of the pretreated pulps compared to the untreated pulp (5.18±0.96 poise).

The optimized physical parameters at which *B. stearothermophilus* xylanase and *C. subvermispora* laccase are the most effective were used for strategies III and IV, involving commercial enzymes. Pretreated agro-residual pulp was further characterized for compositional analysis (Fig. S2 in File S1). Under optimized conditions, the combination of *B. stearothermophilus* xylanase and *C. subvermispora* laccase along with SA was found to be the most effective among all combinations for removal of interfering residual materials (Fig. S2 in File S1).

### Residual laccase activity

**Figure 2:**
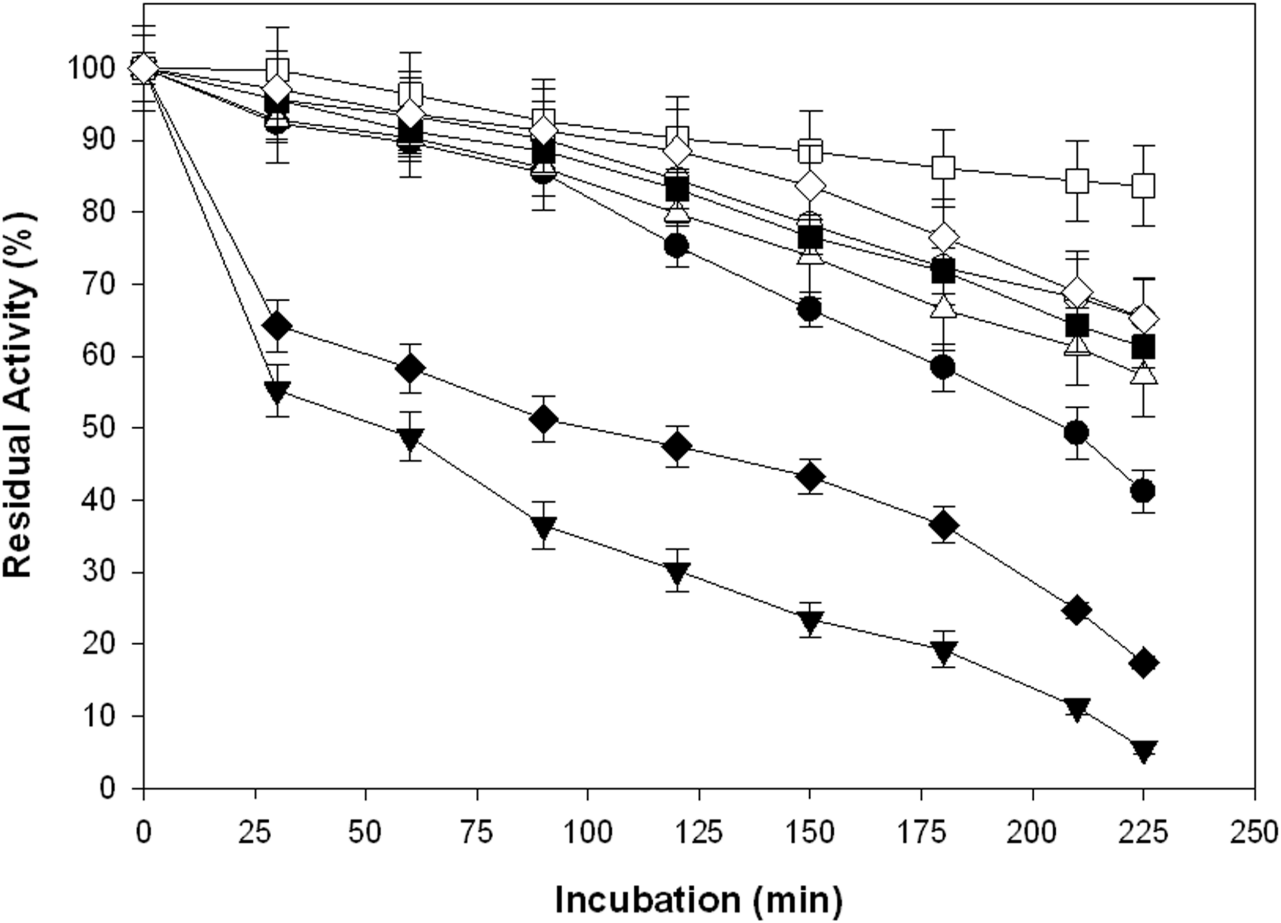
Effect of natural mediator and pulp on the residual activity of laccase during delignification. (•) *C. subvermispora* only; (○) *C. subvermispora* with pulp only; (▾) *C. subvermispora* with SA only; (▵) *C. subvermispora* with pulp & SA; (▪) Commercial laccase only; (□) Commercial laccase with pulp only; (⧫) Commercial laccase with SA only; (⋄) Commercial laccase with pulp & SA.

Both enzyme samples were highly unstable in the presence of SA (natural mediator) as 94.5% and 82.5% of their activity was lost for *C. subvermispora* and commercial laccase, respectively (Fig. 2). However, the presence of pulp stabilized the enzyme samples leading to only 34.8% and 16.3% loss of activity for *C. subvermispora* and commercial laccase, respectively. The pulp in the reaction mixture efficiently prevented denaturation of the enzymes even after the addition of SA to the reaction mixture, resulting in 42.8% and 28.8% loss of activity for *C. subvermispora* and commercial laccase, respectively. A surfactant (Tween-20, Tween-80 and Triton X-100) and a preservative (glycine) were also used to improve the residual activity of *C. subvermispora* laccase. Addition of Tween-20 (1.5%; v/v) provided extra stability to the *C. subvermispora* laccase leading to only 34.4% loss of activity.

### Development of the enzymatic process

With an optimized ED_X_ of 7.5 U per gram of OD pulp at pH 9.0, strategy I, which involved the use of *B. stearothermophilus* xylanase, resulted in a 20.6% reduction in the consumption of Cl_2_ to give brightness identical to that observed for the conventional bleaching process. Strategy II (ED_L_ = 20 U at pH 5.0 and 50°C), which involves the sequential use of *C. subvermispora* laccase resulted in a 30.2% reduction in the consumption of Cl_2_ (Table 3). In order to compare the biobleaching efficiency of *B. stearothermophilus* SDX xylanase (strategy I), commercial Novozyme xylanase (Pulpzyme VLBL) was also used in strategy III, and it resulted in only 17.8% reduction in consumption of Cl_2_. On the other hand, when commercial xylanase and laccase were used sequentially in strategy IV, a reduction of 25.3% in Cl_2_ consumption was observed (Table 3).

**Table 3:** Different schemes for biobleaching agro-residual pulp with a reduced dose of chlorine*.

Parameters	Control	Strategy – I	Strategy – II	Strategy – III	Strategy – IV
	100%^a^	79.4%^b^	69.8%^c^	82.2%^d^	74.7%^e^
**After enzymatic pretreatment stage (X/XL)**					
Brightness (%ISO)	43.6	45.8	46.9	45.2	46.4
Yellowness	15.9	13.9	10.9	14.4	11.7
Whiteness	23.5	24.1	25.4	23.8	24.6
Permanganate no	7.1	6.5	5.3	6.9	5.6
Kappa no	11.5	10.8	9.9	11.2	10.4
**Chlorine, chlorine dioxide treatment stage (C_D_)**					
Total Cl_2_ added (%)	2.50	1.99	1.75	2.06	1.87
Cl_2_:ClO_2_	90∶10	90∶10	90∶10	90∶10	90∶10
Cl_2_ Consumed (%)	2.48	1.62	1.52	1.88	1.61
pH	2.5	2.5	2.5	2.5	2.5
Time (min.)	45	45	45	45	45
Brightness (%ISO)	53.6	53.6	57.2	52.7	55.9
Yellowness	11.6	10.6	9.95	11.2	10.4
Whiteness	8.06	8.41	8.82	8.19	8.27
**Alkali stage (E_OP_)**					
Alkali added (%)	1.8	1.8	1.8	1.8	1.8
Time (min)	120	120	120	120	120
Temp (°C)	70	70	70	70	70
pH	12	12	12	12	12
Brightness (%ISO)	58.4	58.4	59.6	58.2	59.7
Yellowness	9.15	8.85	6.95	9.07	7.92
Whiteness	17.0	18.6	19.5	17.8	18.8
**D_1_ stage**					
ClO_2_ added (%)	0.9	0.678	0.628	0.739	0.672
ClO_2_ consumed (%)	0.806	0.552	0.504	0.627	0.517
pH	3.41	3.25	3.18	3.42	3.35
Time (min.)	180	180	180	180	180
Temp (°C)	70	70	70	70	70
Brightness (%ISO)	84.5	81.6	88.8	80.2	86.1
Yellowness	2.95	2.66	2.05	2.77	2.12
Whiteness	71.5	75.9	73.9	71.6	70.9
**D_2_ stage**					
ClO_2_ added (%)	0.4	0.301	0.279	0.328	0.298
ClO_2_ consumed (%)	0.317	0.263	0.191	0.313	0.211
pH	3.85	3.56	3.37	3.54	3.65
Time (min)	180	180	180	180	180
Temp (°C)	70	70	70	70	70
Brightness (%ISO)	88.1	88.8	89.6	88.6	88.4
Yellowness	1.42	1.27	1.01	1.40	1.11
Whiteness	81.6	82.5	83.2	82.1	82.8
**SO_2_ stage**					
Brightness (%ISO)	88.9	89.4	89.9	89.1	89.2

*Volume of elemental chlorine (Cl_2_) used in control process was considered as 100%.

a: Pulp without pretreatment with 100% Cl_2_; b: Pretreated (with *B. stearothermophilus* xylanase) pulp having 75.4% of Cl_2_; c: Pretreated (with *B. stearothermophilus* xylanase and *C. subvermispora* laccase) pulp having 69.8% of Cl_2_; d: Pretreated (with commercial xylanase) pulp having 82.2% of Cl_2_; e: Pretreated (with commercial xylanase and laccase) pulp having 74.7% of Cl_2._

After the chlorination stage, an alkali has to be added to the reaction mixture before the addition of ClO_2_. The main purpose of alkali addition is to eliminate the loosened and degraded lignin [19]. The addition of ClO_2_ (0.9%) was performed in two steps. An excessive dose of ClO_2_ or the addition of the entire volume of ClO_2_ in a single step may reduce the strength of the pulp fiber [20]. Finally, sulfur dioxide (SO_2_) was added to neutralize the remaining ClO_2_ in the reaction mixture since residual ClO_2_ might otherwise interfere with the final brightness [21]. By following the XC_D_E_OP_D_1_D_2_ bleaching sequence, an increase of 39.8% and 37.8% in the brightness was achieved after the D_1_ stage for strategies I and III, respectively. On the other hand, the XLC_D_E_OP_D_1_D_2_ bleaching sequence resulted in a 48.9% and 44.2% increase in brightness after the same D_1_ stage for strategy II and IV, respectively (Fig. S3 in File S1).

For the analysis of the strength of biobleached pulp, hand sheets (70±1 gm^–2^) were prepared and acclimatized overnight under standard temperature and humidity conditions [22]. Among all the processes, strategy II was the most efficient in improving the strength, while strategy IV was the next most efficient (Table 4). Increases of 13.6% and 12.8% were observed in the tear index (mN m^2^/g) in strategies II and IV, respectively. The improvements in other physical properties such as breaking length and porosity were also observed (Fig. S4 in File S1).

**Table 4:** Effect of enzymatic treatment on different physical properties of the agro-based pulp.

Properties	Control	Strategy-I	Strategy-II	Strategy-III	Strategy-IV
Burst factor	38.1±3.9	40.2±4.4	41.4±4.6	39.8±4.1	41.2±4.3
Burst index (kN/g)	3.72±0.42	3.94±0.41	4.06±0.42	3.90±0.41	4.04±0.47
Tear factor	47.1±4.3	51.8±5.3	52.6±5.5	51.3±4.9	52.2±4.7
Tear index (mNm^2^/g)	4.62±0.61	5.07±0.62	5.15±0.72	5.03±0.32	5.11±0.72
Double fold (no.)	65.0±8.1	74.0±10.1	76.1±11.2	72.2±8.1	76.0±9.1
Tensile strength (Nm/g)	55.3±5.8	57.2±6.1	58.1±5.35	57.1±5.8	57.9±6.2
Breaking length (m)	5640±230	5830±380	5920±420	5820±380	5910±440
Porosity (sec/100 ml)	86.4±8.3	88.3±7.8	89.1±7.9	87.9±8.4	88.9±9.1

### X-ray diffraction (XRD) analysis

The X-ray diffraction analysis of control and enzymatically pretreated wheat straw pulp is shown in Figure S5 in File S1. In all the control and enzymatically pretreated samples, the peak intensity from (002) lattice plane (2θ = 22.3°) represented the crystalline cellulose, while peak intensity of amorphous phase occurred at 2θ = 15.7° [23]. The I_002_ intensities (in cm) of samples pretreated with xylanase produced in the lab and commercial xylanase at 2θ = 22.3° were 5.83 and 5.80, respectively (Table S7 in File S1). For sequentially pretreated substrate at 2θ = 22.3°, the intensities were 9.90 and 9.16 for lab enzymes and commercial enzymes (Table S7 in File S1). Similarly I_am_ intensities (in cm) at 2θ = 15.7° are given in Table S5 of File S1 for individual enzyme pretreated and sequentially pretreated biomasses. XRD analysis revealed that all of the strategies, i.e. individual enzyme pretreatment or sequential enzymatic pretreatment increased the crystallinity index of the pulp; however, the highest improvement in crystallinity was observed in the pretreatment strategy involving xylanase and laccase produced in the lab up to 68.6% (Table S7 in File S1).

### FTIR spectral analysis

FTIR spectra for single enzyme-pretreated and sequentially pretreated pulp samples showed several characteristic and prominent changes (Fig. S6 in File S1). The sequentially pretreated pulp showed absorption structures similar to single enzyme pretreated strategy but with different intensities (Fig. S6 in File S1). The main bands around the region 3,420–3,460 cm^–1^ depict –OH stretching of hydrogen bonding. The increase in their relative intensity after enzymatic treatments is attributed to the increase in cellulosic content of the pulp [24]. The variation in the relative intensity of bands at 2,921–2,917 cm^–1^ and the decrease in the intensity of bands at 607–617 cm^–1^, assigned to CH asymmetrical stretching vibration in CH_3_, CH_2_, and CH, in sequentially pretreated pulp indicated the degradation of aliphatic side chains. The relative intensity around 1,617–1,652 cm^–1^ increased in sequentially pretreated pulp, which is attributed to the release of free carbonyl groups (C = O) due to the action of the enzyme on lignin’s aromatic ring. In single enzyme pretreated pulp, these carbonyl groups remain associated with aromatic rings resulting in lesser absorption. The relative intensity of sequentially pretreated pulp decreased at 1,436–1,416 cm^–1^, a band assigned to aromatic skeletal vibrations combined with –CH_3_ in-plane deformations, showing that some methoxy groups were removed during the enzymatic treatment. The band at 1,320–1,382 cm^–1^ was assigned to aliphatic C–H stretching in CH_3_ (not in –OCH_3_) and phenol-OH group. The decrease in its relative intensity illustrated that either the side chains or phen–OH of lignin decreased after the enzymatic treatment. The decrease in relative intensity at 1,266–1,252 cm^–1^ indicates the degradation of guaiacyl groups. A new band that appeared at 1,737 cm^–1^ in pulp pretreated with lab produced xylanase was assigned to C = O stretching vibration in β-C = O, COOH, and ester, indicating that residual lignin after an enzymatic pretreatment was enriched in these types of functional groups because of the removal of xylan. The changes in pulp crystallinity were derived from A1430/A897 ratio in accordance with the X-ray diffraction studies [24].

### Characterization of effluents

Strategies II and IV resulted in 60.1% and 47.9% reduction in BOD, respectively (Table S8 in File S1), compared to the control process. These results were further supported by the color reduction values. The highest (25.8%) and lowest (12.1%) color reductions were observed with strategies II and III, respectively (Table S8 in File S1). The observed reduction in the color of the effluent was higher than that reported previously, where 11% reduction was reported with Pulpzyme HC [25]. Similarly, 60.1% reduction in BOD was observed with strategy II compared to 23.0% reduction with Kraft pulp in the previous report [26].

### Acute toxicity assay

**Figure 3:**
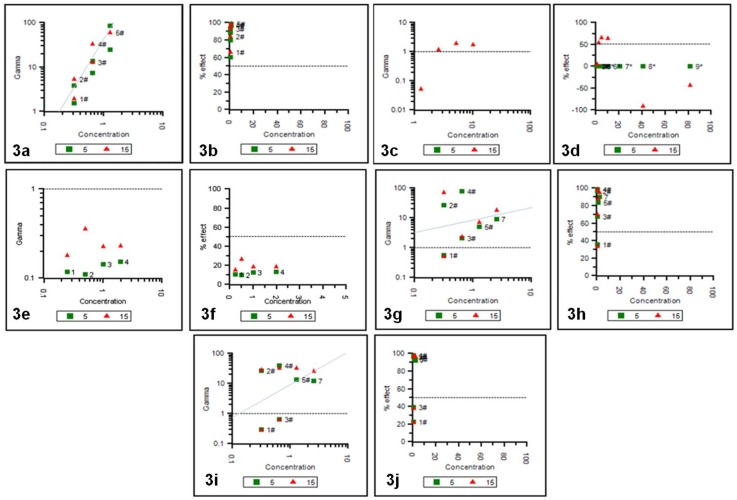
Gamma effect and EC_50_ values for (a, b) control; (c, d) strategy I; (e, f) strategy II; (g, h) strategy III; (i, j) strategy IV. The EC calculated by log linear plotting of Concentration (C) vs. % Light Decrease (%Δ), or more precisely by plotting Gamma (Γ) (which is the corrected ratio of the amount of light lost to the amount of light remaining) versus Concentration.

The chemical intensive control process significantly inhibited the bioluminescence of *Vibrio fischeri* (Fig. 3a, b), as shown by the lower EC*_50–5min_* and EC*_50–15min_* values (Table 5). However, strategy I using *B. stearothermophilus* SDX xylanase more significantly reduced the acute toxicity of the mixed effluent (Fig. 3c, d), as shown by EC_50_ values that were more than double the EC_50_ of the control process (Table 5). Biological processes using Pulpzyme VLBL also reduced the acute toxicity (Fig. 3e, f); however, it was less efficient than the enzyme system produced in the lab (Table 5). Similar TOU (%) results were observed with these enzymes. A 2-fold improvement in TOU (%) was observed for strategy I compared to the control process, whereas the commercial xylanase resulted in 53.6% improvement in TOU (%). A slight increase in acute toxicity was observed with strategies involving the sequential application of xylanase and laccase compared to the single enzyme systems. In fact, each of these strategies alone, i.e., strategy II and IV, was significantly better than the control process in terms of the EC_50_ and TOU (%) values (Table 5). Sequential application of xylanase and laccase produced in lab, reduced the acute toxicity by 43.4–50.0% (Fig. 3 g, h) compared to the control process at different sampling times. In contrast, strategies involving commercial enzymes reduced the acute toxicity by 32.7–38.4% (Fig. 3i, j) compared to the control process at different sampling times (Table 5).

**Table 5:** Reduction in acute toxicity of effluent samples determined through Microtox analyzer and represented by the average values of EC_50_ and unit of toxicity (TOU)*.

Sample	Parameter	EC_50_ (%)	TOU (%)
Control	EC_50–*5min*_	39±4	221±26
	EC_50–*15min*_	32±3	
Strategy I	EC_50–*5min*_	84±9	119±14
	EC_50–*15min*_	82±9	
Strategy II	EC_50–*5min*_	69±7	152±19
	EC_50–*15min*_	64±7	
Strategy III	EC_50–*5min*_	72±7	134±16
	EC_50–*15min*_	69±8	
Strategy IV	EC_50–*5min*_	58±6	166±21
	EC_50–*15min*_	52±6	

*EC_50–*5min*_: determination of inhibition in bioluminescence after 5 min of incubation; EC_50–*15min*_: determination of inhibition in bioluminescence after 15 min of incubation; TOU (%) determination of toxicity unit after 15 min of incubation.

## Discussion

### Model for pulp pretreatment

Owing to the differences in the optimal pH values for the two enzymes and the inhibition of the activity of the mediator system under alkaline conditions, the pulp was subjected to sequential application of xylanase and laccase enzymes. Pretreatment was carried out with xylanase first because the mediators used during laccase pretreatment might generate free radicals capable of hindering the hydrolytic activity of xylanase [18]. Three-dimensional contour plots were also drawn, and it was observed that in xylanase-aided pretreatment, significant decrease in permanganate number was observed (Fig. S7 in File S1). On the other hand, a significant increase in the brightness was observed after the sequential application of enzymes (Fig. S8 in File S1). This might be because all the inhibitory components, which were generated after the use of individual enzymatic pretreatment, were neutralized in the sequential treatment process. Although numerous reports are available on the sequential use of xylanase and laccase for pulp pretreatment [3], [18], this is the first report on the sequential application of xylanase and laccase for biobleaching of the agro-residual pulp.

### Natural mediator and laccase pretreatment

SA was selected as the mediator because of its plant origin and its higher efficiency to decolorize the Azure-B (Fig. S1 in File S1). Further, the improvement observed in the residual activity of the enzyme in the presence of pulp could be due to the cellulosic content of the pulp. Pulp fibers might provide an additional substrate for free radicals generated by the action of the natural mediator. The rate at which these free radicals target the enzyme is decreased owing to the presence of the pulp fiber; therefore, the residual enzyme activity increased. A similar observation was also reported by Fillat et al. [27]. Use of natural mediators will be beneficial in pollution reduction because the higher concentration of free radicals generated due to the action of synthetic mediators that make the whole bleaching process highly toxic and raise environmental concerns [19], [28]. Since commercial laccase has lower specific activity, it was less effective for delignification compared to *C. subvermispora* laccase, which has higher specific activity.

### Properties of pulp obtained from enzymatic pretreatment strategies

Improvement in the strength after enzymatic pretreatment is directly related to the hydrolytic action of xylanase. Hydrolytic enzymes disrupt the surface of cellulosic fiber and generate microfibrils, which in turn lead to a cross-networked, condensed packing of pulp fibers and hence they give extra strength [22]. Since only xylanase has hydrolytic properties, even sequential application of oxidizing enzymes (strategies II and IV) did not improve the strength significantly (Fig. S4 in File S1). Although commercial xylanase alone (Ecopulp, Cartazyme NS-10, and Pulpzyme HC) has been used previously [20], no report is available on the sequential application of xylanase and laccase for processing wheat straw pulp.

Improvement in crystallinity means a decrease in amorphous cellulose and increase in crystalline cellulose of the pulp. The increase in crystallinity might be due to the removal of hemicelluloses and lignin and components adhered to lignin as a result of sequential pretreatment, thereby increasing the cellulose content of the pulp [24]. Sequential pretreatment strategies involve separate laccase supplementation, and this is probably the reason for higher increase in crystallinity in sequential approach than in single enzyme strategy.

### Reduction in pollution load

Achieving less or non-toxic discharge from pulp and paper industry is the biggest challenge in the current scenario. Specific characteristics of the effluents such as BOD and color were determined, and strategy II was found to be the most effective in reducing the pollution load (color), resulting in 25.8% reduction. This might be because of the sequential use of *B. stearothermophilus* SDX xylanase and *C. subvermispora* laccase that removes a significant amount of lignin during pretreatment, which is further removed in the subsequent washes. Washing away the oxidized ligneous material and other hemicellulosic waste reduces the consumption of oxidizing chemicals during bleaching, thereby resulting in effluents with less BOD and colored units. Lesser pollution reduction efficiency of strategy III and IV could be attributed to the presence of surfactants and preservatives in commercial enzyme samples [20]. Media components present in crude enzymes produced in the lab do not interfere with the catalytic properties of the enzymes and hence do not alter their pollution reduction efficiencies [29]. The use of natural mediators in sequential enzymatic approach resulted in better performance in terms of reduction in pollution load. Therefore, the costs associated with the use of natural mediators can be compensated.

### Reduction in acute toxicity

The main reason for the reduced acute toxicity observed with the biological processes was the reduced consumption of oxidizing materials during bleaching. However, a variation was observed in the efficiency of the enzymes produced in lab and commercial enzyme systems. This difference in the efficiency could be explained by the presence or absence of chelators and preservatives. Commercial enzymes contain unidentified preservatives and other compounds to preserve their activity for an extended period of time, and these compounds have been shown to be crucial during the determination of acute toxicity and units of toxicity (http://www.brenntagspecialties. com/en/downloads/Products/Food/Novozymes/Cellulast_1_5L.pdf). Application of commercial enzymes liberates these compounds in the effluent and subsequently increases the biological load and hence the acute toxicity (http://www.sfm.state.or.us/cr2k_subdb/MSDS/CELLUCLAST. PDF). This could explain the lower toxicity of effluents generated using enzymes produced in lab.

Although the sequential application of xylanase and laccase efficiently removes the ligneous material during pretreatment, augmentation of the reaction by mediators caused an increase in the biological load of the effluent generated from these biological processes. Although the mediator was from a natural source, its addition still caused the formation of free radical compounds [13]. Once they formed, they immediately interact with other organic compounds to form highly stable toxicants, which increased the acute toxicity. Because of their natural origin, the concentration of the generated free radical compounds was much less than the intermediates and toxicants generated by the application of synthetic organic mediators [30]. Therefore, application of natural mediator is preferred over synthetic mediator.

## Conclusions

In the current study, sequential biobleaching of agro residual pulp has been proved to be a beneficial strategy compared to the conventional bleaching sequence. Significant reduction in the release of toxic organic compounds was achieved by incorporating sequential enzymatic pretreatment strategy with the conventional bleaching (C_D_E_OP_D_1_D_2_) process. Sequential pretreatment strategy combined with natural mediators further improved the eco-friendly nature of the process. Significant reduction in ecotoxicity also suggests the potential of this approach in the development of green technology for pulp and paper industry.

## Supporting Information

File S1 **Supporting equations, figures, and tables. Equation S1,** Second order polynomial equation showing pretreatment of agro-residual pulp with *B. stearothermophillus* xylanase. A, B, C and D are independent variables corresponds to pH, temperature (°C), enzyme dose (U/mg) and retention time (min), respectively. *Y_1_*, *Y_2_* and *Y_3_* are final response for permanganate number (PN_X_), brightness (B_X_) and yellowness (Y_X_) after pretraetment of agro-residual pulp with *B. stearothermophillus* xylanase. **Equation S2,** Second order polynomial equation showing pretreatment of agro-residual pulp with *C. subvermispora* laccase. where E, F and G are independent variables corresponds to enzyme dose (U/mg), mediator conc. (%) and retention time (min), respectively *Y_4_*, *Y_5_* and *Y_6_* are final response for permanganate number (PN_L_), brightness (B_L_) and yellowness (Y_L_) after pretreatment of agro-residual pulp with *C. subvermispora* laccase. **Figure S1,** Decolorization of Azure-B using natural mediators (syringaldehyde and acetosyringone) and synthetic mediator (1-hydroxybenzatriazole) with *C. subvermispora* and the commercial laccase. *C. subvermispora* laccase with HBT (•), SA (▾), AS (▪); the commercial laccase with HBT (○), SA (▵) and AS (□). **Figure S2,** Effect of different enzymatic treatments on lignin removal. BP: before pulping; AP: after pulping; X: after xylanase treatment; X_L_: after sequential use of xylanase and laccase; X_C_: after treatment with commercial xylanase; X_C_+L_C_: after sequential treatment with commercial xylanase and laccase. **Figure S3,** Brightness (%ISO) value of the agro-residual pulp at different bleaching stages for the different strategies. Enz: after enzymatic pretreatment; C_D_: after chlorination stage; E_OP_: after alkaline peroxide stage; D_1_: after first ClO_2_ stage; D_2_: after second ClO_2_ stage; SO_2_: after final treatment with SO_2_. **Figure S4,** Improvement in the different strength properties of hand sheets made from the agro-residual pulp for various enzymatic treatment strategies. BF: burst factor; BI: burst index (kN/g); TF: tear factor; TI: tear index (mNm^2^/g); DF: double fold (number); TS: tensile strength (Nm/g); BL: breaking length (m); P: porosity (sec/100 ml). **Figure S5,** X-ray diffraction analysis of (a) control sample and enzymatically pretreated sample after (b) strategy I; (c) strategy II; (d) strategy III and (e) strategy IV. **Figure S6,** FTIR spectral analysis of (a) control sample and enzymatically pretreated sample after (b) strategy I; (c) strategy II; (d) strategy III and (e) strategy IV. **Figure S7,** Three dimensional contour plot for the reduction in the permanganate number. *pH and temperature were used as independent variables for xylanase-aided pretreatment. **Figure S8,** Three dimensional contour plot for the increase in the brightness. *enzyme dose and retention time were considered as independent variables for laccase-mediated pretreatment. **Table S1,** Study of interaction among the various nutritional and physical parameters on xylanase production through Plackett-Burman design. ^#^xylanase activity in Unit per gram of dry bacterial bran. *Experiments were performed at pH 8.0. a – Beef extract (%); b – Galactose (%); c – KNO_3_ (%); d – Temperature (°C); e – Incubation time (h); f – Substrate (wheat bran) concentration (%); g – Particle (wheat bran) size (mm); h – Peptone (%); i – Yeast extract (%); j – Dummy variable (blank). **Table S2,** Different levels of variables affecting the xylanase production through CCD-RSM modeling. **Table S3,** Study of interaction among different levels of variables and their effect on xylanase production under SSF. *Xylanase activity in Units per gram of dry wheat bran. a – Peptone (%); b – KNO_3_ (%); c – Temperature (°C); d – Incubation time (h). **Table S4,** List of values of independent variables used for the pretreatment of the agro-residual pulp. **Table S5,** Effect of various temperatures on different properties of wheat straw pulp. *Experimentation was carried out with 15 U/mg of laccase from *C. subvermispora* using 2% of ABTS for 240 min of incubation at pH 5.5. a – temperature in °C; b – permanganate number of pulp; c – Brightness (%ISO) of pulp; d – yellowness (b*) of pulp. **Table S6,** Effect of various pH values on different properties of wheat straw pulp. *Experimentation was carried out with 15 U/mg of laccase from *C. subvermispora* using 2% of ABTS for 240 min of incubation at 50°C. a – permanganate number of pulp; b – Brightness (%ISO) of pulp; c – yellowness (b*) of pulp. **Table S7,** X-ray diffraction analysis of wheat straw pulp after different enzymatic pretreatments. *crystallinity index. X_C_ represents pretreatment with commercial xylanase; X_L_ represent pretreatment with lab-scale xylanase; X_C_+L_C_ represents sequential pretreatment with commercial xylanase followed by commercial laccase; X_L_+L_L_ represents sequential pretreatment with lab-scale xylanase followed by lab-scale laccase. **Table S8,** Characterization of effluents from the entire process for different enzymatic combinations.
(DOC)
