# Supplementary material for: Reduction in Acute Ecotoxicity of Paper Mill Effluent by Sequential Application of Xylanase and Laccase
Source: PLoS One. 2014 Jul 24;9(7):e102581. doi: 10.1371/journal.pone.0102581 (PMC4109937; doi:10.1371/journal.pone.0102581)
Supplement: File S1 — Supporting equations, figures, and tables. Equation S1, Second order polynomial equation showing pretreatment of agro-residual pulp with B. stearothermophillus xylanase. A, B, C and D are independent variables corresponds to pH, temperature (°C), enzyme dose (U/mg) and retention time (min), respectively. Y1, Y2 and Y3 are final response for permanganate number (PNX), brightness (BX) and yellowness (YX) after pretraetment of agro-residual pulp with B. stearothermophillus xylanase. Equation S2, Second order polynomial equation showing pretreatment of agro-residual pulp with C. subvermispora laccase. where E, F and G are independent variables corresponds to enzyme dose (U/mg), mediator conc. (%) and retention time (min), respectively Y4, Y5 and Y6 are final response for permanganate number (PNL), brightness (BL) and yellowness (YL) after pretreatment of agro-residual pulp with C. subvermispora laccase. Figure S1, Decolorization of Azure-B using natural mediators (syringaldehyde and acetosyringone) and synthetic mediator (1-hydroxybenzatriazole) with C. subvermispora and the commercial laccase. C. subvermispora laccase with HBT (•), SA (▾), AS (▪); the commercial laccase with HBT (○), SA (▵) and AS (□). Figure S2, Effect of different enzymatic treatments on lignin removal. BP: before pulping; AP: after pulping; X: after xylanase treatment; XL: after sequential use of xylanase and laccase; XC: after treatment with commercial xylanase; XC+LC: after sequential treatment with commercial xylanase and laccase. Figure S3, Brightness (%ISO) value of the agro-residual pulp at different bleaching stages for the different strategies. Enz: after enzymatic pretreatment; CD: after chlorination stage; EOP: after alkaline peroxide stage; D1: after first ClO2 stage; D2: after second ClO2 stage; SO2: after final treatment with SO2. Figure S4, Improvement in the different strength properties of hand sheets made from the agro-residual pulp for various enzymatic treatment strategies. BF: bu [file pone.0102581.s001.doc]

**Reduction in acute ecotoxicity of paper mill effluent by sequential application of xylanase and laccase**

Saurabh Sudha Dhiman^a,b+^, Gaurav Garg^c,d+^, Jitender Sharma^c^,

Yun Chan Kang^a^, Vipin C. Kalia^e*^, Jung-Kul Lee^a,b*^

^a^ – Department of Chemical Engineering, Konkuk University, 1 Hwayang–Dong, Gwangjin–Gu, Seoul 143–701, Republic of Korea

^b^ – Institute of SK–KU Biomaterials, Konkuk University, 1 Hwayang–Dong, Gwangjin–Gu, Seoul 143–701, Republic of Korea

^c^ – Department of Biotechnology, Kurukshetra University, Kurukshetra – 136 119, Haryana, India

^d^ - Department of Biotechnology, Maharishi Markandeshwar University, Mullana-Ambala – 133 207, Haryana, India

^e^ –Microbial Biotechnology and Genomics, CSIR-Institute of Genomics and Integrative

Biology, Delhi University Campus, Mall Road, Delhi-110007, India

^+^ These authors equally contributed to this work.

*Corresponding Author

E-mail: jkrhee@konkuk.ac.kr, Tel: + Tel.: +82-2-4503505; Fax: +82-2-4583504

E-mail: vckalia@igib.res.in, Tel: + Tel.: +91-11-27666156; Fax: +91-11-27667471

E-mail: yckang@konkuk.ac.kr, Tel: + Tel.: +82-2-20496010; Fax: +82-2-4583504

**Table S1**

| Run | Variables | | | | | | | | | | | Xylanase activity  (U/ g-dbb)^#^ |
| --- | --- | --- | --- | --- | --- | --- | --- | --- | --- | --- | --- | --- |
|  | BE^a^ | G^b^ | K^c^ | T^d^ | IT^e^ | SC^f^ | PS^g^ | P^h^ | YE^i^ | D^j^ | D^j^ |  |
| 1 | 2.00 | 2.00 | 0.5 | 37.0 | 120 | 0.5 | 4.00 | 4.00 | 5.00 | (+) | (–) | 1552±12.32 |
| 2 | 1.00 | 5.00 | 2.00 | 37.0 | 120 | 5.00 | 4.00 | 2.00 | 5.00 | (–) | (–) | 1633±18.76 |
| 3 | 1.00 | 2.00 | 0.5 | 37.0 | 72 | 0.5 | 1.00 | 2.00 | 2.00 | (–) | (–) | 1211±13.55 |
| 4 | 1.00 | 2.00 | 0.5 | 65.0 | 72 | 5.00 | 4.00 | 4.00 | 5.00 | (–) | (+) | 1027±15.67 |
| 5 | 2.00 | 5.00 | 0.5 | 65.0 | 120 | 5.00 | 1.00 | 4.00 | 2.00 | (–) | (–) | 1067±11.69 |
| 6 | 2.00 | 5.00 | 0.5 | 37.0 | 72 | 5.00 | 1.00 | 2.00 | 5.00 | (+) | (+) | 1049±10.75 |
| 7 | 2.00 | 5.00 | 2.00 | 37.0 | 72 | 0.5 | 4.00 | 4.00 | 2.00 | (–) | (+) | 1675±18.94 |
| 8 | 2.00 | 2.00 | 2.00 | 65.0 | 120 | 5.00 | 1.00 | 2.00 | 5.00 | (–) | (+) | 1088±15.74 |
| 9 | 1.00 | 5.00 | 2.00 | 65.0 | 72 | 0.5 | 1.00 | 4.00 | 5.00 | (+) | (–) | 1055±13.75 |
| 10 | 1.00 | 2.00 | 2.00 | 37.0 | 120 | 5.00 | 1.00 | 4.00 | 2.00 | (+) | (+) | 2479±20.22 |
| 11 | 1.00 | 5.00 | 0.5 | 65.0 | 120 | 0.5 | 4.00 | 2.00 | 2.00 | (+) | (+) | 1291±13.54 |
| 12 | 2.00 | 2.00 | 2.00 | 65.0 | 72 | 5.00 | 4.00 | 2.00 | 2.00 | (+) | (–) | 1061±11.62 |

**Table S2**

| Factor code | Factor | Level | | | | |
| --- | --- | --- | --- | --- | --- | --- |
|  |  | –α | –1 | 0 | +1 | +α |
| *X*_1_ | Peptone (%) | 1.00 | 2.00 | 3.00 | 4.00 | 5.00 |
| *X*_2_ | KNO3 (%) | 0.25 | 0.50 | 1.25 | 2.00 | 2.75 |
| *X*_3_ | Temperature (°C) | 23 | 37 | 51 | 65 | 79 |
| *X*_4_ | Incubation time (h) | 48 | 72 | 96 | 120 | 144 |

**Table S3**

| Run | Factors | | | | Xylanase production (U/g-dbb)^*^ | |
| --- | --- | --- | --- | --- | --- | --- |
|  | *X*_1_ | *X*_2_ | *X*_3_ | *X*_4_ | Actual | Predicted |
| 1 | –1 | –1 | –1 | –1 | 1154±112 | 1160±117 |
| 2 | 0 | 0 | 0 | 0 | 1645±135 | 1648±132 |
| 3 | –α | 0 | 0 | 0 | 763±153 | 767±66 |
| 4 | –1 | +1 | +1 | –1 | 2998±201 | 3004±145 |
| 5 | 0 | –α | 0 | 0 | 4048±233 | 4053±223 |
| 6 | 0 | 0 | 0 | 0 | 1645±119 | 1648±138 |
| 7 | +1 | +1 | –1 | –1 | 642±69 | 648±69 |
| 8 | 0 | 0 | 0 | +α | 463±46 | 468±42 |
| 9 | –1 | –1 | +1 | +1 | 309±42 | 307±33 |
| 10 | –1 | –1 | +1 | –1 | 1928±109 | 1934±196 |
| 11 | 0 | 0 | 0 | 0 | 1645±116 | 1648±132 |
| 12 | 0 | 0 | 0 | –α | 1529±177 | 1533±142 |
| 13 | +1 | –1 | +1 | +1 | 853±94 | 549±46 |
| 14 | –1 | +1 | –1 | –1 | 579±69 | 572±59 |
| 15 | +1 | –1 | +1 | –1 | 479±42 | 483±49 |
| 16 | 0 | 0 | 0 | 0 | 1645±115 | 1648±132 |
| 17 | +1 | +1 | –1 | +1 | 1993±205 | 1997±201 |
| 18 | 0 | 0 | –α | 0 | 1493±132 | 1501±233 |
| 19 | +1 | –1 | –1 | –1 | 658±59 | 656±56 |
| 20 | +1 | +1 | +1 | –1 | 573±55 | 575±48 |
| 21 | +1 | –1 | –1 | +1 | 4221±232 | 4235±245 |
| 22 | 0 | 0 | 0 | 0 | 1645±115 | 1648±132 |
| 23 | +1 | +1 | +1 | +1 | 544±49 | 548±52 |
| 24 | 0 | +α | 0 | 0 | 1379±128 | 1376±112 |
| 25 | –1 | +1 | +1 | +1 | 854±69 | 850±79 |
| 26 | –1 | –1 | –1 | +1 | 816±72 | 822±82 |
| 27 | –1 | +1 | –1 | +1 | 2868±181 | 2873±162 |
| 28 | 0 | 0 | 0 | 0 | 1645±115 | 1648±132 |
| 29 | –α | 0 | 0 | 0 | 2161±132 | 2159±132 |
| 30 | 0 | 0 | +α | 0 | 2419±210 | 2423±145 |

**Table S4**

| Factor code | Factor | Level | | | | |
| --- | --- | --- | --- | --- | --- | --- |
|  |  | –α | –1 | 0 | +1 | +α |
| Xylanase aided pretreatment | | | | | | |
| *A* | pH | 7.0 | 8.0 | 9.0 | 10.0 | 11.0 |
| *B* | Temperature (°C) | 50.0 | 55.0 | 60.0 | 65.0 | 70.0 |
| *C* | Enzyme dose (U/ mg) | 2.50 | 5.0 | 7.50 | 10.0 | 12.5 |
| *D* | Retention time (min) | 90 | 120 | 150 | 180 | 210 |
| Pretreatment with laccase mediated system | | | | | | |
| *E* | Enzyme dose (U/ mg) | 10 | 15 | 20 | 25 | 30 |
| *F* | Mediator conc. (%) | 0.5 | 1 | 1.5 | 2 | 2.5 |
| *G* | Retention time (min) | 45 | 90 | 135 | 180 | 225 |

**Table S5**

| T_L_^a^ | PN_L_^b^ | B_L_^c^ | Y_L_^d^ |
| --- | --- | --- | --- |
| 30 | 5.96±0.8 | 46.2±4.2 | 13.6±1.5 |
| 35 | 5.84±0.7 | 46.7±4.8 | 13.2±1.3 |
| 40 | 5.79±0.8 | 46.9±5.2 | 12.9±1.4 |
| 45 | 5.74±0.8 | 47.3±4.5 | 12.6±1.2 |
| 50 | 5.66±0.6 | 47.7±5.3 | 12.4±1.5 |
| 55 | 5.88±0.5 | 47.1±4.9 | 13.1±1.6 |
| 60 | 5.98±0.8 | 46.8±4.8 | 13.4±1.2 |

**Table S6**

| pH | PN_L_^a^ | B_L_^b^ | Y_L_^c^ |
| --- | --- | --- | --- |
| 4.0 | 5.42±0.7 | 46.8±4.8 | 13.2±1.4 |
| 4.5 | 5.32±0.8 | 47.3±5.1 | 12.9±1.5 |
| 5.0 | 5.24±0.5 | 47.9±5.2 | 12.4±1.6 |
| 5.5 | 5.36±0.6 | 47.6±4.9 | 12.6±1.3 |
| 6.0 | 5.40±0.8 | 47.0±5.1 | 13.0±1.3 |

**Table S7**

| Sample | 2θ intensities | | CI* |
| --- | --- | --- | --- |
|  | for crystalline part | for amorphous part |  |
| Control | 3.26 | -- | -- |
| X_C_ pretreatment | 5.80 | 4.6 | 20.6 |
| X_L_ pretreatment | 5.83 | 4.3 | 26.2 |
| X_C_+L_C_ pretreatment | 9.16 | 3.3 | 63.9 |
| X_L_+L_L_ pretreatment | 9.90 | 3.1 | 68.6 |

**Table S8**

| Parameters | Control | Strategy–I | Strategy–II | Strategy–III | Strategy–IV |
| --- | --- | --- | --- | --- | --- |
| BOD (mg/l) | 298±31 | 198±20 | 119±21 | 215±23 | 155±17 |
| Color (mg/l) | 519±55 | 441±45 | 385±33 | 456±39 | 422±36 |

**Figure S1**

**Figure S2**

**Figure S3**

**Figure S4**

**Figure S5**


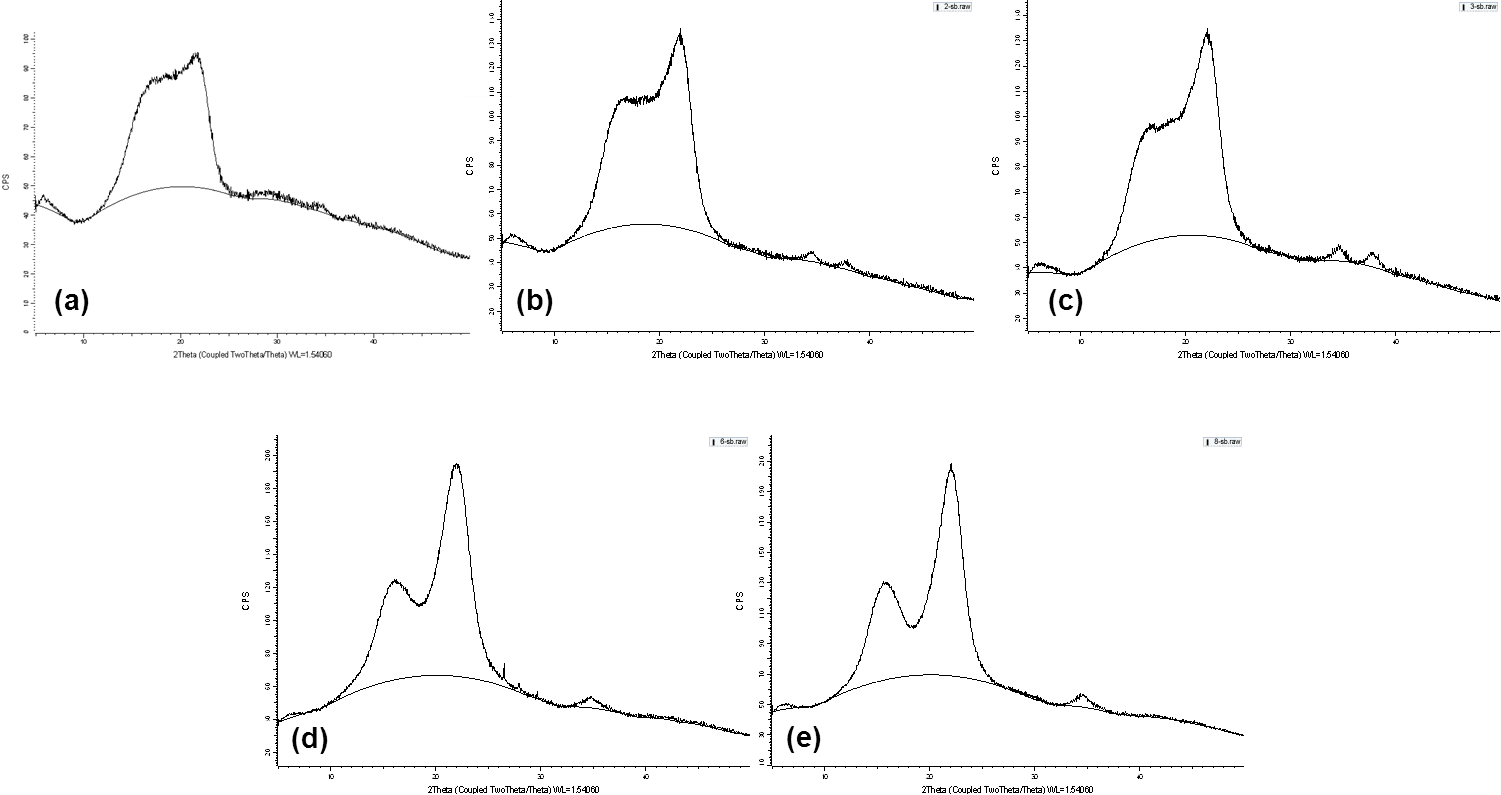


**Figure S6**

**
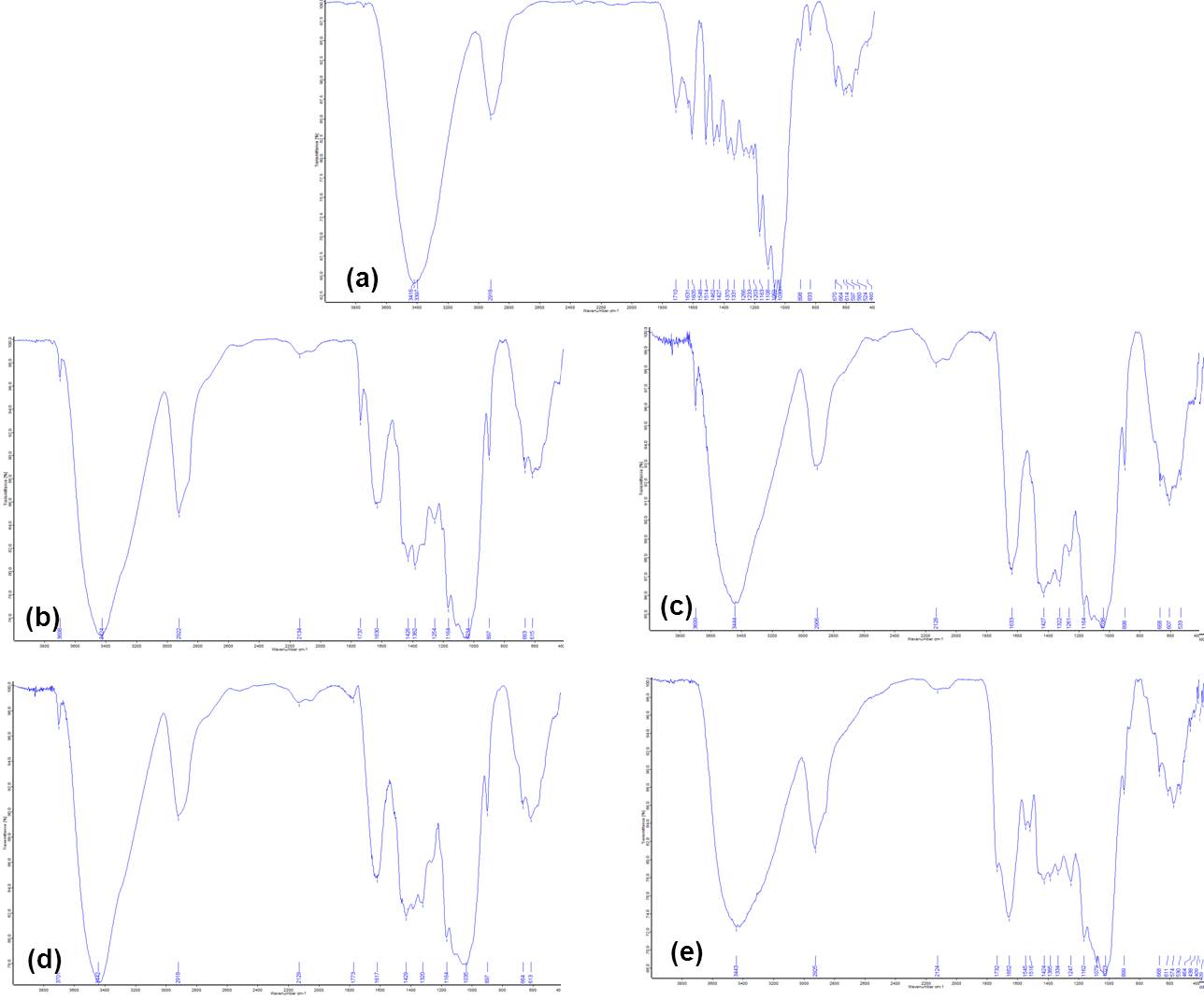
**

**Figure S7**

**
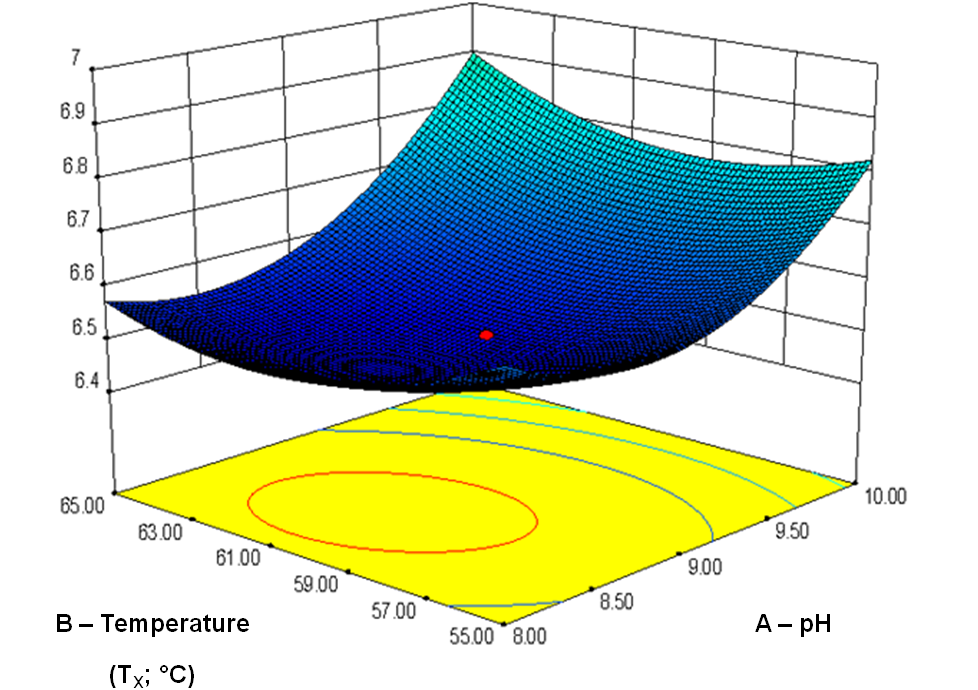
**

**Figure S8**

**
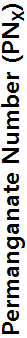

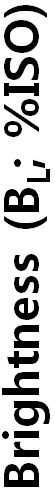
**


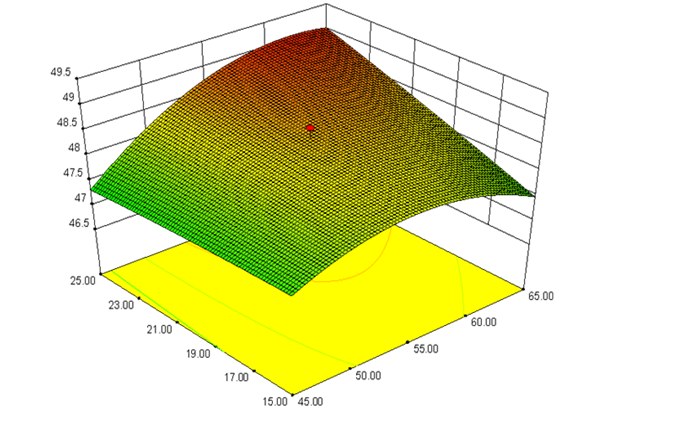


**E – Enzyme Dose**

**(ED_L_; U/ mg)**

**G – Retention Time**

**(RT_L_; min)**

Equation S1

*Y_1_* (PN_X_) = 6.50 + (0.12 × A) + (0.033 × B) – (0.000 × D) + (0.17 × A^2^) + (0.10 × B^2^) + (0.17 × C^2^) + (0.067 × D^2^) + (0.025 × A × B) + (0.012 × A × C) – (0.013 × A × D) – (0.025 × B × C) – (0.025 × B × D) – (0.013 × C × D)

*Y_2_* (B_X_) = 6.77 – (0.27 × A) – (0.19 × B) – (0.095 × C) – (0.15 × D) – (0.15 × A^2^) – (0.11 × B^2^) – (0.16 × C^2^) – (0.096 × D^2^) – (0.25 × A × B) – (0.25 × A × C) – (0.28 × A × D) – (0.24 × B × C) – (0.26 × B × D) – (0.26 × C × D)

*Y_3_* (Y_X_) = 13.95 + (0.30 × A) + (0.026 × B) – (0.11 × C) + (0.016 × D) + (0.30 × A^2^) + (0.036 × B^2^) + (0.13 × C^2^) + (0.076 × D^2^) + (0.028 × A × B) + (0.059 × A × C) + (0.098 × A × D) – (0.042 × B × C) + (0.064 × B × D) – (0.024 × C × D)

Equation S2

*Y_4_* (PN_L_) = 5.24 – (0.34 × E) – (0.22 × F) – (0.23 × G) + (0.09 × E × F) – (0.16 × E × G) – (0.041 × F × G) + (0.10 × E ^2^) – (0.028 × F^2^) + (0.013 × G ^2^)

*Y_5_* (B_L_) = 48.49 + (0.41 × E) + (0.33 × F) + (0.24 × G) + (0.44 × E × F) + (0.21 × E × G) + (0.31 × F × G) – (0.61 × E ^2^) – (0.031 × F ^2^) – (0.60 × G ^2^)

*Y_6_* (Y_L_) = 12.90 – (0.29 × E) – (0.45 × F) – (0.27 × G) + (0.13 × E × F) – (0.30 × E × G) – (0.14 × F × G) – (0.30 × E ^2^) – (0.32 × F ^2^) – (0.36 × G ^2^)
